# Epidemiology of polycystic ovary syndrome: a cross sectional study of university students at An-Najah national university-Palestine

**DOI:** 10.1186/1477-7827-11-47

**Published:** 2013-05-20

**Authors:** Samar Musmar, Asma Afaneh, Hafsa Mo'alla

**Affiliations:** 1 Department of Family Medicine, Faculty of Medicine and Health Sciences, An-Najah National University, P.O. Box 7, 707, Nablus, Palestine; 2 Department of Medicine, Faculty of Medicine and Health Sciences, An-Najah National University, P.O. Box 7, 707, Nablus, Palestine

**Keywords:** Polycystic ovary syndrome, PCOS, Prevalence, Palestine

## Abstract

**Background:**

Polycystic Ovary Syndrome (PCOS) is the most common gynecological endocrinopathy in women of reproductive age. Despite its heavy burden on female reproduction and general health, there is no study regarding PCOS prevalence in Palestine. This study aims to establish prevalence of PCOS among female university students at An-Najah National University-Palestine and to explore its possible risk factors.

**Methods:**

A cross sectional study was conducted on 137 female students using convenience sampling method for age group (18–24) years. PCOS cases were identified according to the National Institute of health (NIH) criteria through clinical interview and assessment for participants at the University clinics. Menstrual irregularities regarding cycle and flow were identified and clinical hyperandrogenism was assessed as the self-reported degree of hirsutism using the modified Ferriman Gallwey (mF-G) scoring method of more than 8 score. Biochemical hyperandrogenism for girls with menstrual irregularities was assessed by measuring free testosterone level. Data were analyzed using SPSS version 17 applying descriptive methods; different risk factor relationships were estimated using bivariate analysis and multivariate logistic regression.

**Results:**

The estimated prevalence of PCOS was 7.3% , acne was the only studied risk factor among others to be statistically significantly related to PCOS patients (OR = 8.430, P-value = 0.015). Clinical Hirsutism was found in 27% of participants, 70% of whom had idiopathic hirsutism.

**Conclusions:**

Prevalence of PCOS in Palestine seems to be relatively high but similar to other Mediterranean statistics. We recommend further studies using wider age group and larger sample for all parts of Palestine in order to generalize results.

## Background

Polycystic ovary syndrome (PCOS) is the most common endocrine disease among women in reproductive age; it is defined as a chronic condition of anovulation or oligo-ovulation with clinical or biochemical hyperandrogenism, which occurs in the absence of any other underlying condition [1]. Women with PCOS are at increased risk of reproductive problems including infertility or subfertility, endometrial cancer and gestational problems. Also may have metabolic disorders including obesity, metabolic syndrome, type 2 diabetes mellitus, dyslipidemia, hypertension and cardiovascular diseases [1].

Until recently, no universally accepted clinical definition existed for PCOS, which remains a syndrome, and as such no single diagnostic criterion is sufficient for clinical diagnosis. The initial National Institute of Health (NIH) diagnostic criteria based on oligomenorrhea, amenorrhea and clinical or biochemical hyperandrogenism were broadened in the 2003 Rotterdam criteria to include PCO findings at ultrasound. More recently the Androgen Excess Society (AES) suggested that the diagnostic criteria should be modified to exclude those with PCO on ultrasound and oligomenorrhea/amenorrhea but no hyperandrogenism [2-4].

Several studies in American and European countries using NIH criteria estimated the prevalence of PCOS at a range of 4-8% [5-11]. Only few studies were done to estimate the prevalence of PCOS in the middle east; two large Iranian studies found prevalence to be around 7% [12,13] , however to the best of our knowledge no study was found to estimate PCOS among Arab women. The objective of this study was to determine the prevalence of PCOS among female university students in Palestine according to the National Institute of health (NIH) criteria and to explore its risk factors.

## Methods

### Study design and the sampling method

A cross-sectional, non-interventional, descriptive and analytical study was conducted during the period of November 2011- March 2012 at An-Najah National University student clinics. Located in Nablus city in the north of West Bank/Palestine, this largest university attracts students from all social classes and from different parts of West Bank. A convenient study sample of 136 participant was calculated using Finite Population Correction formula [14]. Recruitment for the study was done using proper advertisement by posters and student electronic boards targeting all female university students. Of 147 students who agreed to participate, ten (6.8%) students who did not meet inclusion criteria were excluded (found to have hyperprolactinemia, taking oral contraceptives or being pregnant).No one had to be excluded because of other exclusion criteria (using insulin sensitizers, having clinical Cushing disease, or having diabetes).

### Study protocol

During the study period, two trained researchers interviewed recruited participants at the university clinic. After thoroughly explaining the research and its purpose to every participant, a written consent form was obtained followed by completing a checklist datasheet which is based on the inclusion and exclusion criteria about main features of PCOS including menstrual history, hirsutism scoring and some important risk factors. All participants underwent clinical examinations, including body weight, height, waist circumference (WC), and hip circumferences (HC). Waist hip ratio (WHR) was calculated and Body mass index (BMI) was calculated as weight in kilograms divided by the height in meters squared (kg/m2) [15]. BMI values were categorized as underweight (less than 18.5), normal (18.5-24.9), overweight (25–29.9), and obese (equal or more than 30) [15].

Of those who had menstrual irregularities (n = 35 ), 33 agreed to undergo venous blood sampling for hormonal analysis including serum free testosterone (FT), prolactin (PLC) and thyroid stimulating hormone (TSH). Blood samples were centrifuged after 5 minutes of withdrawal, the serum was preserved in iced refrigerator, and then samples were sent daily to a standard diagnostic laboratory in ice bag for analysis.

### Definitions

PCOS diagnosis in this study was defined according to 1990 Consensus of National Institute of Health (NIH) criteria [2] which requires a PCOS case to have oligomenorrhea or amenorrhea, clinical and/or biochemical evidence of hyperandrogenism, and exclusion of other disorders that can result in menstrual irregularities and hirsutism. A participant was considered to have oligomenorrhea if she had less than 8 cycles per year and amenorrhea if she had absence of menses for 6 months or more [1]. This was estimated by recalling menstrual pattern in the last year. Clinical hyperandrogenism was diagnosed by hirsutism assessment using mF-G scoring system figures; we considered the participants to have hirsutism if they scored 8 or more [16]. This cut off point was used by most of the studies especially those done in Mediterranean region or on Caucasian populations. Idiopathic hirsutism (IH) was defined as clinical hirsutism without menstrual irregularities [17]. Biochemical hyperandrogenism was diagnosed by elevated levels of serum FT as it is considered the single most predictive evidence of hyperandrogenemia [18,19].

We excluded Cushing syndrome clinically by the absence of gross clinical features. Serum PLC and TSH were done for all the participants with menstrual irregularities to exclude hyperprolactinemia and thyroid hormone disturbances as other causes of menstrual irregularities.

### Lab measurements

Serum FT level was measured using Gamma counter (Wallac) with a principle of competitive radioimmunoassay; normal values were at a range of 0.2-4.1 pg/ml. TSH test was done using Abbott Axsym- Autommated Immunoassay analyzer and applying ultrasensitive hTSH II which is based on microparticle enzyme immunoassay ; normal values were ranging at 0.39-4.0 mIU/L. PLC values were also measured applying Abbott Axsym- Autommated Immunoassay analyzer which is based on microparticle enzyme immunoassay; normal values ranged between 4.1-28.9 ng/ml. All laboratory tests were done at a standard diagnostic lab which uses internal quality control for all results. The coefficient of variance was 2.5% for PLC, 5% for TSH,and 10% for FT.

### Statistical analysis

Participants were divided into PCOS group and Non-PCOS group. Comparison between the two groups was done applying independent sample T test for continuous variables and Fisher exact Chi square for categorical variables. P values were considered statistically significant at P equal or less than 0.05. Multivariate logistic regression was used to re-examine the significance of the relationship between PCOS and risk factors and to control confounders. Statistical analysis was done using the Statistical Package for Social Sciences (SPSS) version 17.

### Ethical considerations

An-Najah National University IRB Committee approved the study proposal on Nov 20, 2011, and a written informed consent was obtained from every participant. A copy of the written consent is available for review by the Editor-in-Chief of this journal.

## Results

Of 147 who agreed to participate in the study, 137 who met the inclusion criteria were enrolled and completed the study procedure. All the participants were within the age group (18–24) with mean age of 20.2 ± 1.4. Only four of the participants were married and one was divorced; all of which have no fertility problems. About smoking, 27 of the participants reported regular cigarette or water pipe smoking (19.7%). Participants’ weight range was 39.4-82 Kg with the mean of 58.2 ±9.4 kg and the calculated BMI was in the range of 16.8-30.6 and a mean of 22.2 ±3.1.

### The prevalence of polycystic ovary syndrome

Out of 137 study participants, 10 students met the NIH criteria for diagnosis of PCOS making the prevalence rate to be 7.3% (95% CI 4.7-9.9). None of these cases had been diagnosed or treated for PCOS; Table 1 shows that all PCOS cases had oligo-ovulation and clinical hyperandrogenism (100% had Hirsuitism score ≥8, 80% had acne, however none had abnormal serum FT levels). Menstrual irregularities were found in 35 cases of the 137, of which 25 cases had menstrual irregularities without hirsutism. Clinical hirsutism assessed by the mF-G score was found in 38 cases of this study , of whom 28 had idiopathic hirsutism.

**Table 1:** Patients with polycystic ovary syndrome who met individual diagnostic criteria according to the National Institute of Child Health*

**Criteria**	**N (%)**
Clinical and/or biochemical hyperandrogenism	10 (100%)
Hirsutism score ≥8	10 (100%)
Acne	8 (80%)
Increased free testosterone	0 (0%)
Oligo-ovulation	10 (100%)
Chronic oligomenorrhea and/or amenorrhea	10 (100%)
Exclusion of secondary causes	10 (100%)

*Values are raw numbers (percentages).

### Anthropometric characteristics of the sample

There was no significant statistical relation between PCOS group and non-PCOS group regarding weight, WC, WHR and BMI means applying independent samples T test; BMI classes also did not show any significant statistical relationship with PCOS, (Table 2).

**Table 2:** Anthropometric and gynecologic characteristics of the study sample in relation to PCOS

**Anthropometric**	**Total sample**	**Non-PCOS group**	**PCOS group**	P value*
**characteristics**	**(mean ± SD)**	**(mean ± SD)**	**(mean ± SD)**	
Weight(kg)	58.24 ±9.44	58.09 ±9.41	60.19 ±10.14	0.539
Height(cm)	161.83 ±6.17	161.94 ±6.27	160.5 ±4.82	0.394
WC (cm)	80.42 ±7.49	80.45 ±7.51	80.08 ±7.69	0.885
HC (cm)	97.04 ±7.38	96.91 ±7.33	98.62 ±8.16	0.537
W/H ratio	0.83 ±0.047	0.830 ±0.047	0.813 ±0.0516	0.318
BMI(kg/m^2^)	22.2 ±3.13	22.11 ±3.06	23.37 ±3.85	0.333
**BMI classes**	**Total sample N (%)**	**Non-PCOS group N (%)**	**PCOS group N (%)**	P value*
Underweight (less than18.5)	15 (10.9)	14 (11)	1(10)	1.00
Normal (18.5-24.9)	97 (70.8)	91 (71.7)	6(60)	0.47
Overweight (25–29.9)	24 (17.5)	21 (16.5)	3(30)	0.380
Obese (equal or more than30)	1 (0.7)	1 (0.8)	0(0)	1.000
**Gynecologic characteristics**	**Total sample N (%)**	**Non-PCOS group N (%)**	**PCOS group N (%)**	P value*
Hirsutism (mFG more than 8)	38(27.7)	28(22)	10(100)	<0.001
Menstrual irregularities	35(25.5)	25(19.7)	10(100)	<0.001

*P value less than 0.05 was considered statistically significant.

### Risk factors of PCOS

PCOS and non-PCOS groups were studied in relation to many possible risk factors including acne, smoking, presence of frontal baldness, family history of PCOS, menstrual disorders in mothers and hirsutism in mothers. Applying Fisher Exact test, it was found that about 80% of PCOS group had acne and 20% had frontal baldness compared to non-PCOS group (33.9%, and 2.4%) with a significant statistical difference in both cases (Table 3).

**Table 3:** Relationship between possible risk factors and PCOS in the study sample

**Risk Factor**	**Total sample**		**Non-PCOS group**		**PCOS group**		P value*
	**N (%)**		**N (%)**		**N (%)**		
**Acne**	51	37.2%	43	33.9%	8	80%	0.006
**Smoking**	27	19.7%	25	19.7%	2	20%	1.000
**Frontal baldness**	5	3.6%	3	2.4%	2	20%	0.043
**family history of PCOS**	4	2.9%	4	3.1%	0	0%	1.000
**Hirsutism in mother**	17	12.4%	16	12.6%	1	10%	1.000
**Menstrual disorders in mother**	11	8%	10	7.9%	1	10%	0.598

* P value less than 0.05 was considered significant calculated by Fisher exact test.

To adjust for participants’ confounding factors, this relationship was reexamined by using multivariate logistic regression method (Table 4). Family history of PCOS could not be controlled for confounders because there were no cases in the PCOS group with this risk factor. Only acne remained statistically significant (P = 0.017, OR = 8.06, CI: 1.45-44.75); however, frontal baldness became non-significant (P-value = 0.052, OR = 8.16).

**Table 4:** Relationships between PCOS and risk factors after adjustment for the confounding factors

**Risk factor**	**Exp (B)**	**95% CI**	(P-value)*
	**(odds ratio)**	**(lower, upper)**	
**Acne**	8.43	(1.52, 46.82)	0.015
**Frontal baldness**	8.34	(0.98, 70.66)	0.052
**Menstrual irregularities in mother**	1.93	(0.18, 20.75)	0.586
**Hirsutism in mother**	0.67	(0.07, 6.26)	0.727
**Smoking**	1.62	(0.28, 9.14)	0.588

* P value less than 0.05 was considered significant calculated by logistic regression.

### Biochemical features of participants with menstrual irregularities (MI)

Calculated means of FT, TSH and prolactin test results of 33 participants who had menstrual irregularities were analyzed. Although some of the results were abnormal (one case of low TSH and two cases of high prolactin levels), there was no statistically significant relationship of these results to PCOS (Table 5).

**Table 5:** Biochemical features of Menstrual irregularity cases (calculated mean) n = 33

**Lab test**	All MI*	**MI (no PCOS)**	**MI with PCOS**	P value**
	**(mean ± SD)**	**(mean ± SD)**	**(mean ± SD)**	
**FT (conv.)**	0.95 ±0.42	0.98 ±0.39	0.86 ±0.47	0.49
**TSH (SI)**	1.25 ±0.66	1.29 ±0.64	1.16 ±0.73	0.66
**Prolactin**	15.93 ±14.37	17.27 ±16.63	12.87 ±6.62	0.29
**(Conv.)**	(Median 13.4)	(Median 14.1)	(Median 10.4)	

*MI: menstrual abnormalities.

**P value less than 0.05 was considered statistically significant.

## Discussion

The prevalence of PCOS at An-Najah National University in age groups 18–24 years was found to be 7.3%. PCOS prevalence depends on the recruitment process of the study population and criteria used for its definition; using NIH criteria, two Iranian studies found PCOS prevalence to be 7.1% and 7%. The first was done in 2011 on a community sample of age group 18–45 years [12], and the second one was done in 2009 in Isfahan among females referred to the mandatory pre-marriage screening clinic of age 17–34 years [13]. Shared common ethnic and sociodemographic factors between Iranian and Palestinian women might explains this similarity in PCOS prevalence in addition to using the same criteria of diagnosis in the study despite the differences in age group and sampling methods. Using same criteria for diagnosis in other parts of the world found a close range of PCOS prevalence; examples are Australia 8.7%, Spain, 6.5%, Greek Island of Lesbos 6.7%, the southeastern United States 4%, and Sweden 4.8% [5-9]. Clinical hirsutism in this study was found in 27% of participants (Table 2), about 70% of whom had idiopathic hirsutism; these women cannot be entirely excluded from the diagnosis of PCOS, because they may have been oligoovulatory, despite their reported regular episodes of vaginal bleeding.

It is expected that we will have double the prevalence of PCOS if we use Rotterdam criteria for diagnosis [13]; although this is a newer criteria for diagnosis, many authors argue that its use may overestimate the prevalence of PCOS. Disagreement has persisted regarding whether the two additional phenotypes (oligo-ovulation with PCO or hyperandrogenism with PCOS) in Rotterdam criteria actually do have classical PCOS, because PCO on ultrasound is a very common finding in normal population [20]. Also in some studies they found that using NIH criteria predicts the metabolic risk more appropriately than Rotterdam criteria [21].

When we explored acne as possible risk factors for PCOS [22], 80%. of PCOS group were found to have acne, and having acne was found to increase risk to develop PCOS by eight times (Tables 3 and 4). This is consistent with findings of Turkish and American PCOS studies where women with PCOS had acne at a rate of 53% [23,24].

Although we found frontal baldness in 20% of PCOS group, this was not statistically significant after adjustment for confounders (Table 4). Frontal baldness is usually found in disorders associated with higher levels of androgen such as androgen secreting tumors [25]. This is supported by our finding of all PCOS group to have normal FT serum level (Table 5).

Smoking, history of hirsutism and history of irregular menses in mothers were found to be insignificantly related to the presence of PCOS (Tables 3 and 4). These finding are shared by some studies [5] but not by others [26]; differences in population characteristics, methodology and sample size might explain these finding differences. Of those who had menstrual abnormalities there was no statistical biochemical difference between those who met the definition of PCOS or not (Table 5). Although none of PCOS cases in our study had high serum FT , clinical hirsutism was significant in all cases using mF-G score (mean11.8). This is in agreement with Haung etal [19] who found that supra-normal levels of FT were present in 57.6% of PCOS patients diagnosed by NIH criteria. The main strength of this study is that being the first PCOS prevalence study in Palestine, and it touches very important women health issue. With the international criteria of diagnosis used in this study, very good results about prevalence and risk factors were drawn that further studies can be built on.

## Conclusions

PCOS prevalence is relatively high among young Palestinian women; the prevalence is similar to Mediterranean and Caucasian populations but higher than that of south East Asia. Further larger studies in Palestine and Arab countries are needed to improve diagnosis and control of this common women health problem.

## Competing interests

The authors declare that they have no competing interests.

## Authors’ contribution

SM participated in the study design and coordination of the study protocol. AA and HM worked on preparing the background and literature review and performed the field work of data collection in addition to statistical analysis.SM reviewed the statistical analysis and drafted the results and discussion. AA and SM drafted the manuscript and all three authors reviewed and approved it.
